# Pregnancy Rates after Hysteroscopic Endometrial Polypectomy versus Endometrial Curettage Polypectomy: A Retrospective Study

**DOI:** 10.3390/medicina59101868

**Published:** 2023-10-20

**Authors:** Mikiko Nishioka, Tadashi Maezawa, Hiroki Takeuchi, Katsuyuki Hagiwara, Sachiyo Tarui, Mito Sakamoto, Erina Takayama, Hideaki Yajima, Eiji Kondo, Hiroaki Kawato, Hiroyuki Minoura, Ken Sugaya, Aisaku Fukuda, Tomoaki Ikeda

**Affiliations:** 1 Department of Obstetrics and Gynecology, Graduate School of Medicine, Mie University, 2-174 Edo-bashi, Tsu, Mie 514-8507, Japan; m-nishioka@med.mie-u.ac.jp (M.N.); h-takeuchi@med.mie-u.ac.jp (H.T.); mito-s@med.mie-u.ac.jp (M.S.); h-yajima0119@med.mie-u.ac.jp (H.Y.); eijikon@med.mie-u.ac.jp (E.K.); t-ikeda@med.mie-u.ac.jp (T.I.); 2 Department of Obstetrics and Gynecology, Mie University Hospital, 2-174 Edo-bashi, Tsu, Mie 514-8507, Japan; erina-t@med.mie-u.ac.jp; 3 Center of Advanced Reproductive Medicine, Mie University Hospital, 2-174 Edo-bashi, Tsu, Mie 514-8507, Japan; 4 Faculty of Education, Mie University, 1577 Kurima-Machiya-cho, Tsu, Mie 514-8507, Japan; hagi@edu.mie-u.ac.jp; 5 Department of Obstetrics and Gynecology, IVF Osaka Clinic, 1-1-14 Nagatahigashi, Higashiosaka, Osaka 577-0012, Japan; tarui@ivfosaka.com (S.T.); fukuda@ivfosaka.com (A.F.); 6 Department of Obstetrics and Gynecology, Kawato Ladies Clinic, 1-16-11 Betsumei, Yokkaichi, Mie 510-0007, Japan; hk593@hotmail.co.jp; 7 Department of Obstetrics and Gynecology, Minoura Ladies Clinic, 3-9-17 Isoyama, Suzuka, Mie 510-0256, Japan; hminoura76@gmail.com; 8 Department of Obstetrics and Gynecology, Saiseikai Matsusaka General Hospital, 15-6 asahimachiichiku, Matsusaka, Mie 515-8557, Japan; k.sugaya@mac.com

**Keywords:** endometrial polypectomy, pregnancy, IVF-ET, snare, curettage

## Abstract

*Background and Objectives*: A relationship between endometrial polypectomy and in vitro fertilization (IVF) pregnancy outcomes has been reported; however, only a few studies have compared polyp removal techniques and pregnancy rates. We investigated whether different polypectomy techniques with endometrial curettage and hysteroscopic polypectomy for endometrial polyps affect subsequent pregnancy outcomes. *Materials and Methods*: Data from 434 patients who had undergone polypectomy for suspected endometrial polyps using transvaginal ultrasonography before embryo transfer in IVF at four institutions between January 2017 and December 2020 were retrospectively analyzed. Overall, there were 157 and 277 patients in the hysteroscopic (mean age: 35.0 years) and curettage (mean age: 37.3 years) groups, respectively. Single-blastocyst transfer cases were selected from both groups and age-matched to unify background factors. *Results*: In the single-blastocyst transfer cases, 148 (mean age: 35.0 years) and 196 (mean age: 35.9 years) were in the hysteroscopic and curettage groups, respectively, with the 148 cases matched by age. In these cases, the pregnancy rates for the first embryo transfer were 68.2% (odds ratio (OR): 2.14) and 51.4% (OR: 1.06) in the hysteroscopic and curettage groups, respectively; the resulting OR was 2.03. The pregnancy rates after up to the second transfer were 80.4% (OR: 4.10) and 68.2% (OR: 2.14) in the hysteroscopic and curettage groups, respectively, in which the OR was 1.91. The live birth rates were 66.2% (OR: 1.956) and 53.4% (OR: 1.15) in the hysteroscopic and curettage groups, respectively, in which the odds ratio was 1.71. These results show the effectiveness of hysteroscopic endometrial polypectomy compared to polypectomy with endometrial curettage. No significant difference was found regarding the miscarriage rates between the two groups. *Conclusions*: Hysteroscopic endometrial polypectomy resulted in a higher pregnancy rate in subsequent embryo transfer than polypectomy with endometrial curettage. Therefore, establishing a facility where polypectomy can be performed hysteroscopically is crucial.

## 1. Introduction

Endometrial polyps, which cause irregular bleeding and infertility, occur in 7.8–34.9% of women [1] and reportedly in 15–24% of infertile patients [2,3]. They are localized, adherent, or stalked projections of the uterine mucosa that result from abnormal hyperplastic growth of the glands and stroma around the vascular core [4,5]. Histologically, an endometrial polyp comprises endometrial glands and stroma around the vascular axis of the spiral artery [6]. Although estrogen receptors are overexpressed in the glandular epithelium of polyps [7] and exposure has been suggested to be involved in the etiology of endometrial polyps [8], the exact etiology of endometrial polyps has not been determined.

Endometrial polyps are categorized as macropolyps (visible on transvaginal ultrasonography) and micropolyps (1–2 mm in diameter and recognized hysteroscopically). Micropolyps have been reported to coexist with chronic endometritis at a high rate [9] and are effectively treated with antibiotics [10]. Treatment with antibiotics also dramatically improves in vitro fertilization (IVF) pregnancy rates by approximately 65% [11,12,13]. However, most infertile patients with macropolyps develop endometritis, and polypectomy has been reported to improve endometritis [2]. Additionally, the pregnancy rate after macropolypectomy is reportedly 23–65% [14,15,16], and polypectomy is expected to improve pregnancy rates.

Endometrial polypectomy is performed using endometrial curettage under anesthesia. However, because endometrial curettage involves the non-visual excision of polyps using placental forceps or curettes, potential damage to endometrial tissue other than the polyps exists, and some patients may experience a decreased pregnancy rate postoperatively due to endometrium thinning [17]. Therefore, the hysteroscopic resection of confirmed polyps has recently been widely used. Various hysteroscopic methods have been reported, including excision using a snare [18], a slender resectoscope [19], and a transvaginal morcellator [20]. Compared to curettage, which removes many endometrial tissues, hysteroscopic resection only partly resects endometrial polyps. However, snare resection has drawbacks, such as the time required to learn the technique and the installation and running costs of the slender resectoscope and transvaginal morcellator. Therefore, if the main focus is on pregnancy rates, the effects and benefits are significant and can be expected to increase pregnancy rates.

Hysteroscopy before IVF is expected to increase pregnancy outcomes [21]. Specifically, performing hysteroscopy in the cycle preceding the ovarian stimulation cycle could improve IVF outcomes in asymptomatic patients with normal transvaginal ultrasound findings undergoing their first IVF attempt. A previous review revealed that clinical pregnancy and live birth rates were higher in the hysteroscopy group than in the controls [21].

To begin with, uterine abnormalities can cause increased miscarriage rates because of the poor implantation environment and their impact on the developmental environment of the fetus. Appropriate uterine surgery has been reported to result in high pregnancy rates and favorable birth rates [22]. Therefore, minimally invasive surgery, including laparoscopic surgery, for infertile patients is expected to improve favorable IVF outcomes and pregnancy rates without IVF [23]. Surgery has recently become increasingly minimally invasive, and many techniques have proven to be relatively effective and safe [24]. Therefore, surgical treatment should be considered an option for organic abnormalities that cause infertility.

Infertility frequently results from uterine or ovarian disease without symptoms in infertile patients, and abnormalities may be noted for the first time during infertility testing. The prevalence of unsuspected intrauterine abnormalities identified at hysteroscopy in the asymptomatic IVF population is as high as 50% [25,26,27], and their early detection would likely contribute to improved pregnancy outcomes. Therefore, reproductive facilities should incorporate more hysteroscopies as a tool to detect the causes of infertility early.

Although a relationship between endometrial polypectomy and IVF pregnancy outcomes has been reported, only a few studies have compared polyp removal techniques and pregnancy rates. Therefore, this study aimed to retrospectively compare IVF outcomes after non-visual polypectomy with endometrial curettage and hysteroscopic polypectomy and evaluate the results with respect to the surgical method of choice.

## 2. Materials and Methods

### 2.1. Ethical Approval

This retrospective observational study was approved by the Ethics Committee of Mie University Hospital (approval number: H2021-104), and information was provided to patients through an opt-out method. The Ethics Committee waived the need for informed consent since opt-out consent was deemed acceptable.

### 2.2. Study Population

Patients with suspected endometrial polyps on transvaginal ultrasonography before embryo transfer in IVF who had undergone polypectomy at four facilities (Mie University Hospital, IVF Osaka Clinic, Saiseikai Matsusaka General Hospital, and Minoura Ladies Clinic) between January 2017 and December 2020 were included in this study.

### 2.3. Study Design

This was a retrospective observational study. Patients with endometrial polyps scheduled for IVF at the four facilities were included during the study period. The inclusion criteria included patients who were diagnosed with endometrial polyps before IVF, who had undergone endometrial polypectomy, who had subsequently undergone IVF embryo transfer, and whose subsequent progress was confirmed in their medical records. The exclusion criteria included patients who had not undergone embryo transfer after endometrial polyp excision and those whose progress after embryo transfer could not be confirmed.

**Figure 1:**
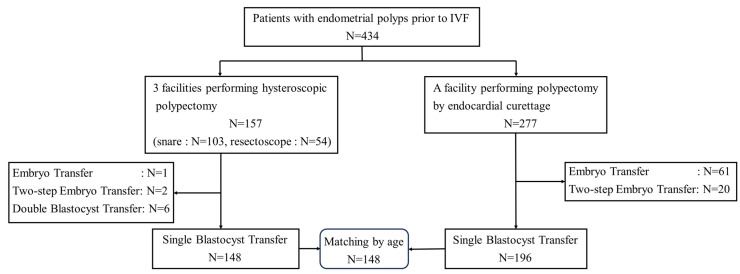
There were 434 cases with polyps before in vitro fertilization (IVF) and 157 with hysteroscopic polypectomy. Of these, 103 and 54 cases were resected using a snare and resectoscope, respectively. Polypectomy with endometrial curettage was performed in 277 cases. Single-blastocyst transfer was performed in 148 hysteroscopic and 196 endorectal curettage cases. Among these, 148 were matched by age.

Three facilities performed hysteroscopic polypectomy for endometrial polyps. Based on polyp size and location findings, some cases were resected through snare polypectomy using a flexible speculum, while others were resected without energization using a resectoscope. Endometrial polyps were excised at one facility using endometrial curettage after they were identified through hysteroscopy (Figure 1). The primary endpoints were pregnancy, miscarriage, and live birth rates after polypectomy. After the pregnancy rates of all embryo transfers were evaluated, single-blastocyst transfer cases were re-evaluated. Pregnancy rates were first evaluated for patients who became pregnant during the first embryo transfer, and those who did not were followed up until the second embryo transfer. Patients who became pregnant during the second embryo transfer and those who became pregnant during the first embryo transfer were collectively evaluated as patients who became pregnant up to the second embryo transfer. Single-blastocyst transfers were matched by age to eliminate the effects of age, and pregnancy, miscarriage, and live birth rates were evaluated after polypectomy. As a secondary endpoint, the endometrial thickness in each group was evaluated after polypectomy.

### 2.4. Surgical Procedure

**Figure 2:**
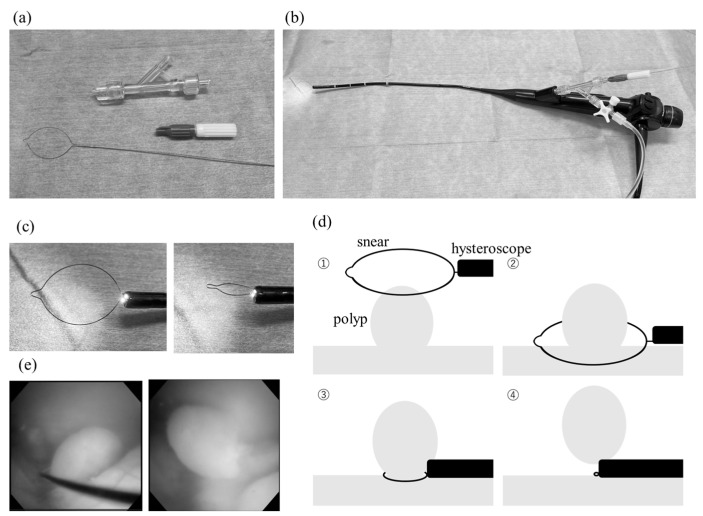
(**a**) Lin snare^®^, which consists of a wire with a looped end, a tube that passes water from the side, and a device that holds the wire in place; (**b**) hysteroscope with snare attached; (**c**) changes with the snare in and out; (**d**) schematic of polypectomy with snare: the ring portion of the wire is hooked around the base of the polyp, and the wire is pulled with the instrument at hand to reduce the size of the ring and remove the polyp; (**e**) use of snare: on the left is where the wire is hooked up, and on the right is how the polyp is removed.

At one institution, polyp excision was performed using endometrial curettage after confirming the presence and location of the polyps using a flexible hysteroscope (curettage group). A flexible hysteroscope is a flexible endoscope with a camera (Figure 2b) whose tip can be moved up and down manually [28]. It allows for observation of the uterus while refluxing water through the uterus and does not require cervical dilation because of its narrow tip. For endometrial curettage, after confirming the polyp’s location using a hysteroscope, the cervix was dilated using a Hegar dilator, and curettage was performed using placental forceps or curettage either blind or while confirming through transabdominal ultrasound. Finally, the procedure was terminated after confirmation via hysteroscopy.

At the remaining three institutions, after the polyps were identified using a flexible hysteroscope, the polyp stem was removed using a snare and subsequently removed from the body. Alternatively, the polyp was identified using a resectoscope, and a loop or ball electrode was used without energization to remove the polyp, which was subsequently removed from the body (hysteroscopic group). The snares were made using the Lin snare system^®^ (Hakko Medical Co., Tokyo, Japan) by inserting a wire with a looped tip into the threaded portion of a flexible hysteroscope. Next, the polyp was removed by squeezing the loop after hooking it at its base and moving the wire back and forth while passing water from the side of the wire. A resectoscope uses a 9 mm rigid speculum to remove lesions in the uterus by moving a loop- or ball-shaped instrument back and forth using a hand-held control [29]. Additionally, the instrument is connected to an electrode and energized to perform incision and coagulation. The diameter of the camera is large, and the procedure requires cervical dilation and anesthesia. In this study, the electrodes were not connected when the resectoscope was used; therefore, the polyps were excised bluntly. Finally, the excised polyps were looped around the polyp’s largest diameter and placed on the body with the hysteroscope and polyp in close contact (Figure 2).

### 2.5. Embryo Transfer after Endometrial Polypectomy

Embryo transfer after polypectomy was performed according to each institution’s endometrial adjustment method (hormone replacement or natural cycle). It was usually performed within at least two cycles after treatment. If the first embryo transfer did not result in a pregnancy, the embryos were transferred in successive cycles or one cycle apart. For cases where the first pregnancy was successful but resulted in miscarriage, the next embryo transfer was performed after the second menstrual period. Embryos that were transferred during the second time point after polypectomy were included in this study. The transferred embryos could be either embryos or blastocysts. Additionally, the transfer method is independent of whether it involves a single-embryo (blastocyst), double-embryo (blastocysts), or two-step embryo (transfer of an embryo and a blastocyst in the same cycle) transfer. However, only single-blastocyst transfers were analyzed after all cases were evaluated. Transfers were performed on embryos that were grade III or higher according to Veeck’s classification, which is an index used to prioritize the transfer of split-stage embryos. The uniformity of the blastomere and the number and rate of fragments determine the grade (Figure S1). Blastocysts were transferred from viable blastocysts with an inner cell mass (ICM) grade of C or higher according to the Gardner classification, which is an index used to prioritize the transfer of blastocysts. Furthermore, the degree of blastocyst expansion and the number and rate of ICM and trophectoderm cells determine the grade (Figure S1).

### 2.6. Statistical Analysis

The data are presented as means. R software was used to analyze the data. The Mann–Whitney U test was used to examine the differences in mean age and endometrial thickness between the two groups. Regarding clinical pregnancy, miscarriage, and live birth rates, the two-proportion Z-test (pooled) was used to compare items. The significance level was set at <5%. In this study, single-blastocyst transfer cases were selected for background uniformity, and subsequently, matching by confounding factors was performed to derive test results that excluded confounders, if any, between each group for the present results. For matching, one-to-one nearest neighbor matching based on Euclidean distance was employed, and the post-matching samples were tested for differences in proportions (using normal approximation) between each group, with the significance level set at <5%.

## 3. Results

### 3.1. Pregnancy, Miscarriage, and Live Birth Rates from Embryo Transfer after Endometrial Polypectomy

Table 1 summarizes the number of cases, age, endometrial thickness, and clinical outcomes after polypectomy in each group for all cases. Table 1 also includes the *p*-values of the statistical tests. During the study period, 434 patients with endometrial polyps were scheduled for IVF at the four facilities. The number of patients in the hysteroscopic group was 157, with a median age of 35 (24–46) years. Of these, 103 and 54 cases were resected using snare polypectomy and Trans Cervical Resection (TCR), respectively. Additionally, the number of patients in the curettage group was 277. Specifically, the patients in the curettage group were significantly older than those in the hysteroscopic group, with a median age of 37 (25–47) years. Of the cases where a single-blastocyst transfer was performed, 148 and 196 were hysteroscopic and endometrial curettages, respectively (Figure 1).

The initial endometrial thicknesses after polypectomy were 11.0 and 11.2 mm in the hysteroscopic and curettage groups, respectively, both in the 11 mm range, although the endometrium was significantly thicker in the curettage group than in the hysteroscopic group.

One (0.6%), two (1.3%), and six (3.8%) cases of single-embryo, double-embryo, and two-step embryo transfers, respectively, were performed in the hysteroscopy group. In the curettage group, 61 (22.0%) embryo and 20 (7.2%) two-step embryo transfers were performed. The pregnancy rate for the first embryo transfer in the hysteroscopic group (67.5%) was significantly higher than that in the curettage group (42.6%). In contrast, the pregnancy rate after up to the second embryo transfer was higher in the hysteroscopy group (81.5%) than in the curettage group (55.2%). The live birth rate in the hysteroscopy group (65.0%) was higher than that in the curettage group (40.1%). However, no significant difference was found in the miscarriage rates between the two groups (Table 1).

### 3.2. Pregnancy, Miscarriage, and Live Birth Rates for the Single-Blastocyst Transfer Only

Table 1 presents the results for the cases with single-blastocyst transfer after endometrial polypectomy. Regarding the single-blastocyst transfer after endometrial polypectomy, the median ages in the hysteroscopic and curettage groups were 35 (24–46) years in 148 patients and 36 (25–45) in 196, respectively, indicating that the curettage group was significantly older than the hysteroscopic group.

The initial endometrial thicknesses after polypectomy were 11.0 and 11.4 mm in the hysteroscopic and curettage groups, respectively, suggesting that the endometrium was thicker in the curettage group than in the hysteroscopic group; however, both were within the 11 mm range.

The pregnancy rate in the first embryo transfer in the hysteroscopic group (68.2%) was significantly higher than that in the curettage group (52.0%). In contrast, the pregnancy rates after up to the second attempts were 80.4% and 65.3% in the hysteroscopic and curettage groups, respectively, signifying that they were higher in the hysteroscopic group than in the curettage group. The live birth rates were 66.2% and 49.5% in the hysteroscopic and curettage groups, respectively, with a higher rate in the hysteroscopic group than in the curettage group. However, no significant difference was found in the miscarriage rates between the two groups (Table 1).

### 3.3. Pregnancy, Miscarriage, and Live Birth Rates after Matching by Confounding Factors

This study’s results showed that the pregnancy rate was significantly higher in the hysteroscopic group than in the curettage group; however, the age of the hysteroscopic group was lower than that of the curettage group. Since pregnancy rates are generally higher in younger women, the effect of age on each of these results is discussed below.

In the single-blastocyst transfer cases, the standardized mean difference (SMD) was 0.459 for patients who became pregnant and those who did not after the first embryo transfer (mean age: 34.6 and 36.6 years). The SMDs were 0.729 (mean age: 34.6 and 37.6 years, 0.846 (mean age: 34.0 and 37.3 years), and −0.398 (mean age: 36.8 and 35.2 years) for patients who became pregnant and those who did not up to the second transfer, patients with and those without live births, and patients with and those without miscarriages, respectively (Table S1). Since the absolute values of SMDs were all >0.1, which was the standard for significant differences, the pregnancy, live birth, and miscarriage rates were considered to be influenced by age. Particularly, the finding that younger age groups are more likely to conceive and have live births was valid. However, the mean ages were 34.8 and 35.9 years for the hysteroscopic and curettage groups, respectively, with an SMD of 0.261, indicating an age bias between the groups. Therefore, since age was likely to be a confounding factor when considering the association between the method of polypectomy and pregnancy, miscarriage, and live birth rates, matching by age was performed between the hysteroscopic and curettage groups for single-blastocyst transfer cases.

For the single-blastocyst transfer cases with matching by age, the number of cases, age, endometrial thickness, and clinical outcomes after polypectomy in each group are summarized in Table 2. In the single-blastocyst transfer cases, the sample size after matching was 148 cases (sample size of the hysteroscopic group), and the SMD after matching was 0.067, suggesting that the age bias among the groups was almost eliminated. In the hysteroscopic group, the pregnancy rate after the first embryo transfer and that after up to the second embryo transfer were 68.2% and 80.4%, respectively; the miscarriage rate was 16.2%, and the live birth rate was 66.2%. In contrast, the pregnancy rate after the first embryo transfer and that after up to the second embryo transfer in the curettage group were 51.4% and 68.2%, respectively; the miscarriage rate was 15.5%, and the production rate was 53.4%. Both pregnancy and live birth rates were significantly higher in the hysteroscopic group, whereas no significant differences were found in the miscarriage rates (Table 2).

## 4. Discussion

Endometrial polyps are a relatively frequent infertility disorder, recently linked to chronic endometritis [20]. Polypectomy is frequently performed when small polyps are suspected to cause implantation failure [13]. It was previously performed non-visually under anesthesia using placental forceps or curettes and hysteroscopically using a resectoscope for large polyps. However, both procedures require anesthesia and are burdensome for patients. Additionally, non-visual endometrial curettage frequently involves the curettage of normal endometrial tissue around the polyp, which may result in infertility in some cases because of endometrial thinning-induced embryo implantation failure [30]. Polypectomy using a snare has been reported to have minimal impact on the endometrial tissue [17]. A snare is considered to have little effect on the endometrium because only polyps can be removed using a hysteroscope with visualization of the inside of the uterus. However, only a few reports have compared subsequent pregnancy outcomes in patients treated with curettage and hysteroscopic polypectomy.

In this study, we compared the postoperative IVF outcomes between patients in whom endometrial curettage was performed after polyps were identified using a flexible hysteroscope and those in whom endometrial polyps were removed using a hysteroscope. Single-blastocyst transfer cases were also extracted and examined to unify the background factors. Because a significant difference was found in age between the groups in these results, matching by age was performed to remove age bias. The pregnancy rate was significantly higher in patients who had undergone hysteroscopic resection of endometrial polyps than in those who had undergone curettage.

Endometrial polyps have a high recurrence rate [18], and embryo transfer should be performed immediately after polypectomy. Therefore, pregnancy rates after the second embryo transfer were used for comparison in this study. The pregnancy rates were 80.4% and 68.2% in the hysteroscopic and curettage groups, respectively, when matched by age, with the hysteroscopic group having a significantly higher pregnancy rate than the curettage group (Table 2). This may be due to a certain degree of removal of normal endometrial tissue through curettage. Since no difference was found between the two groups regarding the miscarriage rate, the polypectomy technique appeared to have no effect.

A relationship between endometrial thickness and pregnancy rate has long been reported [31], and endometrial curettage reportedly causes endometrium thinning [32,33]. Cases with an endometrium of <7 mm did not result in pregnancy [34]. In this study, the endometrial thickness before embryo transfer was greater in the curettage group than in the hysteroscopic group, although both groups were in the 11 mm range, and neither group showed obvious endometrium thinning; therefore, the effect of polypectomy on endometrial thickness was considered low. However, differences were observed in the pregnancy rates during embryo transfer after polypectomy, possibly due to inflammation or other effects of the endometrium caused by endometrial curettage. Intrauterine adhesions caused by endometrial curettage or infection have been suggested to cause infertility [35]. If the basal layer of the endometrium is damaged during curettage, inflammation is triggered during the wound-healing process [36,37]. Patients with healed chronic endometritis have higher pregnancy and live birth rates than those with persistent inflammation [11]; therefore, healed inflammation in the uterus is important for improving pregnancy rates. These events may prevent embryo implantation by affecting endometrial formation.

Although there are various devices and techniques for hysteroscopic endometrial polypectomy, performing endometrial polypectomy using these methods is useful for early pregnancy in infertile patients. Hysteroscopic endometrial polypectomy, which has less impact on the endometrium, is a useful procedure that leads to earlier patient pregnancy, as suggested by this study’s results. We examined cases at multiple institutions because of the limited number of cases at one institution. However, biases, such as differences in polypectomy techniques and embryo transfer methods at each institution, could not be eliminated. Additionally, perinatal risks, such as diabetes, hypertension, and obesity, were excluded from this study; therefore, including these factors in future analyses is necessary.

## 5. Conclusions

This study showed that hysteroscopic endometrial polypectomy resulted in a significantly higher pregnancy rate than endometrial curettage polypectomy.

## Figures and Tables

**Table 1:** Comparison of results between the hysteroscopic and curettage groups in endometrial polypectomy and the effect of age on this data. (**a**) All cases. (**b**) SBT cases.

Variable	Hysteroscopic Group (n = 157)	Curettage Group (n = 277)	*p*-Value
Age [years], median (range)	35 (24–46)	37 (25–47)	<0.0001 *
Endometrial thickness [mm], median (range)	11.0 (6.0–19.0)	11.2 (7.4–19.1)	0.0278 *
Clinical pregnancies/first embryo transfer, frequency [%]	106 (67.5)	118 (42.6)	<0.0001 †
Clinical pregnancies/up to the second embryo transfer, frequency [%]	128 (81.5)	153 (55.2)	<0.0001 †
Miscarriages, frequency [%]	26 (16.6)	42 (15.2)	0.7002 †
Live births, frequency [%]	102 (65.0)	111 (40.1)	<0.0001 †
**Variable**	**Hysteroscopic group** **(n = 148)**	**Curettage** **group** **(n = 196)**	***p*-value**
Age [years], median (range)	35 (24–46)	36 (25–45)	0.0211 *
Endometrial thickness [mm], median (range)	11.0 (6.0–19.0)	11.4 (7.4–17.6)	0.0260 *
Clinical pregnancies/first embryo transfer, frequency [%]	101 (68.2)	102 (52.0)	0.0025 †
Clinical pregnancies/up to the second embryo transfer, frequency [%]	119 (80.4)	128 (65.3)	0.0021 †
Miscarriages, frequency [%]	24 (16.2)	32 (16.3)	0.9781 †
Live births, frequency [%]	98 (66.2)	97 (49.5)	0.0019 †

Data are reported as mean and (range) for age and endometrial thickness, and as frequency and (percentage) for the other variables. * Mann–Whitney’s test; † two-proportion Z-test. SBT: single-blastocyst transfer.

**Table 2:** Tests for differences between proportions and ratios in the SBT group matched by age.

Variable	Hysteroscopic Group (n = 148)	Curettage Group (n = 148)	*p*-Value
Age [years], median (range)	35 (24–46)	35 (25–45)	0.6049 *
Endometrial thickness [mm], median (range)	11.0 (6.0–19.0)	11.2 (7.4–17.6)	0.1093 *
Clinical pregnancies/first embryo transfer, frequency [%]	101 (68.2)	76 (51.4)	0.0030 †
Clinical pregnancies/up to the second embryo transfer, frequency [%]	119 (80.4)	101 (68.2)	0.0166 †
Miscarriages, frequency [%]	24 (16.2)	23 (15.5)	0.8736 †
Live births, frequency [%]	98 (66.2)	79 (53.4)	0.0243 †

Data are reported as mean and (range) for age and endometrial thickness, and as frequency and (percentage) for the other variables.* Mann–Whitney test; † two-proportion Z-test.

## Data Availability

The data supporting the results of this study are available from the corresponding author upon reasonable request.

## Supplementary Materials

The following supporting information can be downloaded at: https://www.mdpi.com/article/10.3390/medicina59101868/s1, Table S1: Study of the effect of age on pregnancy, live birth, and miscarriage rates in each of the outcomes of single-blastocyst transfer; Figure S1: Veeck and Gardner classifications.
