# Supplementary material for: Pregnancy Rates after Hysteroscopic Endometrial Polypectomy versus Endometrial Curettage Polypectomy: A Retrospective Study
Source: Medicina (Kaunas). 2023 Oct 20;59(10):1868. doi: 10.3390/medicina59101868 (PMC10608414; doi:10.3390/medicina59101868)
Supplement: Supplementary file 1 [file medicina-59-01868-s001.zip › medicina-2602303-supplementary.pdf]

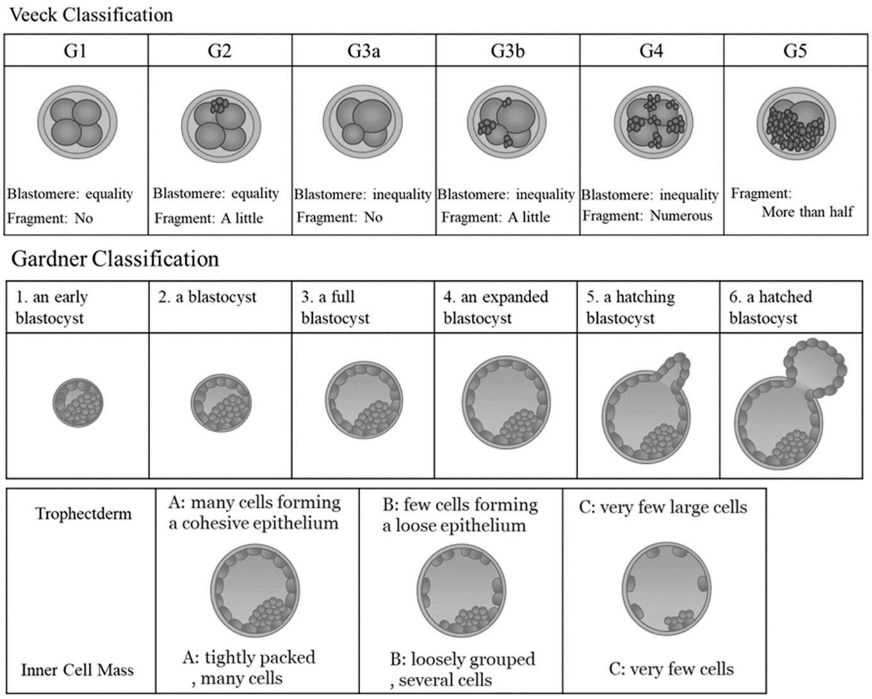

Figure S1. Veeck and Gardner classifications. Veeck Classification: Grading is performed based on the blastomeres' uniformity and the amount of fragments, as shown in the figure. Gardner classification: The percentage of blastocyst formation is determined, and each blastocyst is assigned a score using this classification. Briefly, blastocysts were given a numerical score from 1 to 6 based on their degree of expansion and hatching status, as follows: 1, an early blastocyst with a blastocoel that is less than half of the embryo's volume; 2, a blastocyst with a blastocoel that is half of or greater than half of the embryo's volume; 3, a full blastocyst with a blastocoel that is filling the embryo; 4, an expanded blastocyst with a blastocoel volume larger than that of the early embryo, with a thinning zona; 5, a hatching blastocyst with the trophectoderm starting to herniate through the zona; and 6, a hatched blastocyst, where the blastocyst has completely escaped from the zona. For blastocysts graded as 3–6 (i.e., full blastocysts onward), the inner cell mass development was assessed as follows: A, tightly packed, many cells; B, loosely grouped, several cells; or C, very few cells. The trophectoderm was assessed as follows: A, many cells forming a cohesive epithelium; B, few cells forming a loose epithelium; or C, very few large cells.

Table S1. Study of the effect of age on pregnancy, live birth, and miscarriage rates in each of the outcomes of single blastocyst transfer.

Data Basis	Pregnancy+	Pregnancy-	SMD
Age of cases at first embryo transfer	34.6	36.6	0.459
Age of cases with embryo transfer up to the second	34.6	37.6	0.729
**Data Basis**	**Live birth acquisition cases**	**No live birth acquisition cases**	**SMD**
Age of cases with embryo transfer up to the second	34.0	37.3	0.846
**Data Basis**	**Miscarriage case**	**No miscarriage cases**	**SMD**
Age of cases with embryo transfer up to the second	36.8	35.2	−0.328

SMD (Standardized Mean Difference) of age in single blastocyst transfer cases.
